# A systematic review of the effectiveness of mental health promotion interventions for young people in low and middle income countries

**DOI:** 10.1186/1471-2458-13-835

**Published:** 2013-09-11

**Authors:** Margaret M Barry, Aleisha M Clarke, Rachel Jenkins, Vikram Patel

**Affiliations:** 1 WHO Collaborating Centre for Health Promotion Research, National University of Ireland Galway, University Road, Galway, Ireland; 2 WHO Collaborating Centre for Research and Training for Mental Health, Institute of Psychiatry, King’s College London, 16 De Crespigny Park, London SE5 8AF, UK; 3 Centre for Global Mental Health, London School of Hygiene & Tropical Medicine, Keppel Street, London WC1E 7HT, UK and Sangath, Goa, India

**Keywords:** Mental health promotion, Young people, Low and middle income countries, Systematic review

## Abstract

**Background:**

This systematic review provides a narrative synthesis of the evidence on the effectiveness of mental health promotion interventions for young people in low and middle-income countries (LMICs). Commissioned by the WHO, a review of the evidence for mental health promotion interventions across the lifespan from early years to adulthood was conducted. This paper reports on the findings for interventions promoting the positive mental health of young people (aged 6–18 years) in school and community-based settings.

**Methods:**

Searching a range of electronic databases, 22 studies employing RCTs (N = 11) and quasi-experimental designs conducted in LMICs since 2000 were identified. Fourteen studies of school-based interventions implemented in eight LMICs were reviewed; seven of which included interventions for children living in areas of armed conflict and six interventions of multicomponent lifeskills and resilience training. Eight studies evaluating out-of-school community interventions for adolescents were identified in five countries. Using the Effective Public Health Practice Project (EPHPP) criteria, two reviewers independently assessed the quality of the evidence.

**Results:**

The findings from the majority of the school-based interventions are strong. Structured universal interventions for children living in conflict areas indicate generally significant positive effects on students’ emotional and behavioural wellbeing, including improved self-esteem and coping skills. However, mixed results were also reported, including differential effects for gender and age groups, and two studies reported nonsignficant findings. The majority of the school-based lifeskills and resilience programmes received a moderate quality rating, with findings indicating positive effects on students’ self-esteem, motivation and self-efficacy. The quality of evidence from the community-based interventions for adolescents was moderate to strong with promising findings concerning the potential of multicomponent interventions to impact on youth mental health and social wellbeing.

**Conclusions:**

The review findings indicate that interventions promoting the mental health of young people can be implemented effectively in LMIC school and community settings with moderate to strong evidence of their impact on both positive and negative mental health outcomes. There is a paucity of evidence relating to interventions for younger children in LMIC primary schools. Evidence for the scaling up and sustainability of mental health promotion interventions in LMICs needs to be strengthened.

## Background

Mental health is fundamental to good health and wellbeing and influences social and economic outcomes across the lifespan [1-3]. Childhood and adolescence are crucial periods for laying the foundations for healthy development and good mental health. It is estimated that 10-20% of young people worldwide experience mental health problems [4]. Poor mental health in childhood is associated with health and social problems such as school failure, delinquency and substance misuse, and increases the risk of poverty and other adverse outcomes in adulthood [3]. Interventions that promote positive mental health equip young people with the necessary life skills, supports and resources to fulfill their potential and overcome adversity. Systematic reviews of the international evidence, which come predominantly from high income countries (HICs), show that comprehensive mental health promotion interventions carried out in collaboration with families, schools and communities, lead to improvements not only in mental health but also improved social functioning, academic and work performance, and general health behaviours [5-13].

Despite the recognition of the importance of mental health promotion for children and adolescents, mental health remains a neglected public health issue, especially in low and middle-income countries (LMICs). Mental health is inequitably distributed as people living in poverty and other forms of social disadvantage bear a disproportionate burden of mental disorders and their adverse consequences [14-17]. There is increasing recognition of the relevance of mental health to global development strategies, and in particular to the achievement of the Millennium Development Goals (MDGs), including improving child and maternal health, universal education, combating HIV/AIDS and other diseases, and eradicating poverty [18,19]. As 90% of the world’s children and adolescents live in LMICs, where they constitute up to 50% of the population [20], there is an urgent need to address the mental health of young people as part of the wider health promotion and development agenda.

Schools are one of the most important community settings for promoting the mental health of young people [21]. The school setting provides a forum for promoting emotional and social competence as well as academic learning and offers a means of reaching the significant number of young people who experience mental health problems [22-25]. Educational opportunities throughout life are associated with improved mental health outcomes. The promotion of emotional health and wellbeing is a core feature of the WHO’s Health Promoting Schools initiative [26]. There is good evidence that mental health promotion programmes in schools, especially those adopting a whole school approach, lead to positive mental health, social and educational outcomes [13,27-29]. Programmes incorporating life skills, social and emotional learning and early interventions to address emotional and behavioural problems, produce long-term benefits for young people, including improved emotional and social functioning, positive health behaviours, and improved academic performance [5,13,25,27-31]. To date there has been comparatively little research on school and community-based mental health promotion interventions for young people in LMIC settings and no systematic attempt to synthesize the evidence from such settings. This is the goal of this paper. The work described here was undertaken in 2011–2012 as part of the World Health Organization Task Force on Mainstreaming Health Promotion. Established on foot of the WHO 7^th^ Global Conference on Health Promotion [32], the Task Force sought to develop a package of evidence-based health promotion actions addressing priority public health conditions in LMICs.

The objectives of the review were:

•To synthesize evidence on the effectiveness of mental health promotion interventions for young people that have been implemented in LMICs.

•To identify gaps in the existing evidence and highlight areas where further research is needed.

## Methods

### Study selection

This systematic review conforms to the guidelines outlined by the PRISMA 2009 checklist. A research protocol for the original review was agreed with the Members of the WHO Task Force and the Cochrane Public Health Group (CPHG). Studies were eligible for inclusion if the intervention was designed to promote positive mental health for young people in LMIC settings. For the purpose of this review, mental health promotion interventions were defined as any planned action, programme or policy, which was undertaken with the aim of improving mental health or modifying its determinants. Evidence in relation to the studies for young people aged 6–18 years across all school and community settings was included, with no exclusions based on gender or ethnicity. Academic and grey literature published from 2000 onwards in printed or electronic format was deemed eligible for inclusion. In order to include studies of comparable quality, we considered study designs including randomized controlled trials, cluster randomized controlled trials, and quasi-experimental study designs. The primary outcomes of interest were mental health and wellbeing benefits including; indicators of positive mental health such as self-esteem, self-efficacy, coping skills, resilience, emotional wellbeing; negative mental health such as depression, anxiety, psychological distress, suicidal behaviour; and wellbeing indicators such as social participation, empowerment, communication and social support. Secondary health related outcomes were also noted. Studies with the following characteristics were excluded from the review; (i) selective and indicated prevention interventions, as defined by Mrazek and Haggerty [33], (ii) studies with no control/comparison group, and (iii) qualitative only studies.

### Search strategy

Academic databases including PubMed, PsychInfo, Scopus, ISI Web of Knowledge, Cochrane database of systematic reviews were searched. Health Promotion and Public Health Review databases were also searched including Evidence for Policy and Practice information and Coordinating (EPPI) Centre; University of York National Health Service Centre for reviews and dissemination; National Institute of Clinical Excellence (NICE); Effective Public Health Practice, Health Evidence Canada; WHO programmes and projects. Additional sources included Google Scholar and reference list of relevant articles, book chapters and reviews. Key individuals and organizations identified through the search process were contacted to identify further details on publications. The electronic search strategy used across all databases is provided in Table 1. The last search for the original systematic review of mental health promotion interventions was completed on 11^th^ March 2011 and included articles published between January 2000 - December 2010. A repeated search was conducted on 7th September 2012 to update results and included articles published between January 2011 – June 2012.

**Table 1:** Original search strategy for electronic databases (+ denotes terms used for updated search in Sept 2012)

**Mental health terms**	**Origin**	**Population**	**Setting**	**Intervention terms**	**Related outcomes**
Mental health +	Middle income country+	Infant	Home	Promotion+	Gender
Psychosocial+	Low income country+	Child+	Pre-school+	Prevention+	Child health
Wellbeing+	Developing world+	Adolescent+	School+	Intervention+	Stigma+
Lifeskills+	Developing country+	Young people+	Classroom+	Program+	Discrimination
Empowerment+		outh+	Community+	Policy+	Primary care
Mental capital+		Adult	Out-of-school+	Implementation +	Maternal health
Resilience+		Worker	Health service	Evaluation+	Violence+
Social emotional+		Employee	Workplace	Home visiting	Sexual health +
Mental health literacy+		Family		Early years	HIV prevention+
		Indigenous community		Parenting	Social capital
		Population group		Organizational	Social networks
					Social functioning
					Microfinance+
					Micro-credit+

Searches included: (* denotes multiple word endings including singular and plural).

Mental health terms AND Origin AND Population AND Intervention Terms.

e.g. mental health OR psychosocial OR wellbeing OR lifeskills OR empowerment OR mental capital OR resilience OR social emotional OR mental health literacy

AND

middle income countr* OR low income countr* OR developing world OR developing countr*

AND

child* OR adolescent* OR young people OR youth

AND

Promotion OR prevention OR intervention OR program* OR policy OR implementation OR evaluat*.

Mental health terms AND Origin AND Population AND Setting.

Mental health terms AND Origin AND Population AND Related Outcomes.

Mental health terms AND Origin AND Setting AND Related Outcomes.

Mental health terms AND Origin AND Related outcomes AND Intervention Terms.

Mental health terms AND Origin AND Related outcomes AND Setting.

Mental health terms AND Origin AND Related outcomes AND Population.

### Study selection and data collection

Using the search strategy described above, all titles and abstracts retrieved were scanned for relevance. Duplicates, articles not relevant, and articles that did not meet the inclusion criteria were removed. Full text papers were obtained for studies that were selected for inclusion. Studies were subsequently selected relating to young people and were classified according to (i) school-based programmes (ii) community-based programmes for adolescents. Two reviewers assessed the studies in order to ensure that they met the inclusion criteria set out for this review.

### Data analysis

As the interventions and outcomes evaluated in the included studies were too diverse to allow a quantitative synthesis of the study findings, a narrative synthesis was undertaken. Following the guidelines of the Cochrane Public Health Group, the methodological quality of the intervention evaluations was assessed using the Quality Assessment Tool for Quantitative Studies developed by the Effective Public Health Practice Project [34]. Studies were assessed for selection bias, study design, confounders, blinding, data collection and withdrawals and drop-outs. Each study was rated independently by two reviewers (MB and AC). The quality assessments were compared and disagreements were resolved through discussion. Based on the ratings of each of the six components, each study received an overall global rating of strong, moderate or weak. Following the quality assessment stage, the inclusion of studies and extraction of key findings was finalized. Extracted data were entered into a table of study characteristics (Table 2) including the quality assessment ratings for each study.

**Table 2:** Summary characteristics of included studies

**Study Name, Country, Study author**	**Target Group**	**Type of Intervention & Duration**	**Implementation Issues**	**Study design**	**Outcomes** *	**Quality Assessment**	
**Life Skills education programme**[35] India *Srikala & Kumar, 2010*	Youth (14–16 years) in secondary schools	Lifeskills education intervention. Skills taught include critical thinking, decision making, problem solving, communication, and coping skills Implemented once a week, (1 hr) over 12 to 20 sessions during one academic year Sessions taught by class teacher	Programme content and materials based on needs assessment with students, parents, NGOs and policy makers Teachers trained over 3 days	Quasi-Experimental - random selection of schools with matched control design: N = 1028 adolescents Control received standard civic education classes	Significant improvement in: - self-esteem, - perceived self-efficacy - pro-social behavior - perceived adequate coping Participants had significant: - better adjustment with teachers - better adjustment in school - improved classroom behaviour No change in adjustment with parents and peers	Moderate	
**School based physical fitness programme**[36]Santiago, Chile * Bonhauser et al., 2005*	Secondary school students age 15 years in low socioeconomic area in Chile	School based physical fitness Four units made up of three sessions each week (90 min each) for ten weeks for each unit Sessions taught by regular teachers	Teachers and students designed intervention	Quasi-experimental design N = 198 students from high school Students in control group received 90 minute exercise class once a wee	Significant improvement in adolescents’: - anxiety scores - self-esteem scores No significant changes in depression scores Significant increases in physical fitness: - oxygen capacity - speed and jump performance scores	Moderate	
			Involvement of the Board of Directors was viewed as essential in order to incorporate intervention into curriculum activities.				
**HealthWise Program**[37,38] Cape Town, South Africa *Smith et al., 2008; Caldwell et al., 2010*	Secondary school students grades 8–9 (mean of 14 years) in low income township in Cape Town	School based leisure, life skills and sexuality education intervention 12 lessons provided in grade 8 followed by 6 booster sessions in grade 9 Programme delivered by class teacher.	Cultural adaptation of the TimeWise programme [39] and Botvin’s Lifeskills programme [40] Schools with greatest involvement in teacher training and implementation reported more positive outcomes on intrinsic student motivation.	Quasi-experimental N = 2193 adolescents mean age 14 years Life Orientation curriculum taught in control schools	Significant - increase in intrinsic motivation - decrease in introjected motivation and amotivation Increase in perception of condom availability in intervention group Control group had ‘steeper increase’ in recent and heavy use of alcohol and cigarette use. Programme effects on alcohol, cigarette use greater for girls.	Moderate	
**Resiliency Programme**[41] South Africa *de Villiers & van den Berg, 2012*	Children age 11–12 in Grade 6 in middle-class suburbs of South Africa	Resiliency intervention provided 15 sessions on promoting emotional regulation, stress management, interpersonal skills and problem solving. Each session lasted 90 minutes and delivered over three weeks	Parents and teachers were not involved with the programme	Solomon Four Group Design N = 161 children age 11–12 years from four schools Waitlist control Three month follow up	Significant improvement in - interpersonal strength - emotional regulation - self appraisal - emotional reactivity Improved self appraisal scores maintained at three months follow up. No significant improvement in - family involvement - intrapersonal strength - school functioning - affective strength - sense of mastery - sense of relatedness - family appraisal - general social support.	Weak	
**Resourceful Adolescent Program – (RAP-A) Depression Prevention Programme**[42] Mauritius *Rivet-Duval et al., 2011*	Secondary school students age 12–16 years in Mauritius	Universal depression prevention programme includes cognitive behavioural and interpersonal approaches 11 one hour weekly sessions with 8–12 participants per group. Teachers implemented sessions	RAP-A is an Australian evidenced based intervention [43]. Details of cultural adaptations not reported Teachers attended two day training workshop involving 16 hours of training, received ongoing support and half day booster training session 6 months post initial training	RCT N = 160 students from two single sex secondary schools age 12–16 years Waitlist control Six months follow-up	Significant improvement in - depressive symptoms - hopelessness - self esteem - coping skills. Improvements in self esteem and coping skills maintained at 6 months follow up. Improvements in depression symptoms and hopelessness not maintained at 6 months follow up.	Strong	
**Make a Difference (MAD) about Art: Community-Based Art Therapy Intervention**[44] Nekkies township, South Africa *Mueller et al., 2011*	Children affected by HIV and AIDS age 8–18 in deprived community in South Africa	Community based psychosocial intervention which consisted of art education activities designed to build a sense of self-worth, self-concept, empowerment and emotional control Children attended sessions for six months. Programme implemented in school by trained ‘Youth Ambassadors’ (youth workers)	Sessions were led by team of trained and supervised ‘youth ambassadors’ Being violent towards others and witnessing violence in the home were key predictors of self-efficacy	Quasi-experimental N = 297 youth age 8–18 years from one school	Significant programme effect on self-efficacy scores No programme effect on: - depression scores - emotional and behavioural scores - self esteem scores	Moderate	
**Peer-support group intervention for AIDS orphans**[45] Uganda *Kumakech et al., 2009*	Children age 10–15 years reported to have lost one or both parents due to AIDS	Peer-support intervention aims to encourage participants to reflect, challenge and face difficult experiences and to develop coping skills Two peer support exercises held per week in classroom for 10 weeks. Teachers trained to deliver intervention	Peer-group support exercises were originally intended for adults [46] and were modified for children	Cluster-randomized control trial N = 326 children age 10–15 from 20 schools	Significant reduction in: - anxiety scores - depression scores - anger scores No significant reduction effect on self-concepts	Strong	
**Classroom based psychosocial intervention (CBI)**[47] Nepal *Jordans et al., 2010*	Children affected by armed conflict age 11 – 14 years	School based psychosocial intervention aims to reduce distress and increase resilience and empowerment through enhancing coping, pro-social behaviour 15 sessions delivered over course of 5 weeks. Delivered by para-professionals.	Intervention was developed by Centre for Trauma Psychology in Boston Interventionists from targeted communities were selected and trained over 15 days Counsellor provided regular supervision	Cluster randomised controlled trial N = 325 students age 11–14 years from 8 schools Waitlist control	No significant effect on social emotional wellbeing Significant gender effects including: - reductions in general psychological difficulties and aggression for boys - increased pro-social behaviour for girls Significant increase in sense of hope for older children	Strong	
**Classroom-Based psychosocial Intervention (CBI)**[48] Palestine *Khamis et al., 2004*	Children and adolescents affected by armed conflict aged 6–11 and 13–16 years	School based psychosocial intervention aims to reduce distress and increase resilience and empowerment (same as intervention above) Programme implemented by trained CBI counselors	Recommendations: - provide booster training to CBI interventionists - organize monthly group meetings among intervention coordinators to assure fidelity of the interventionists and to address ongoing technical issues that arise	RCT N = 664: - 406 children age 6–11 years - 258 adolescents, age 13–16 years] Waitlist control	Intervention group had significantly: - better attributional style - reduced level of self-blame - higher perceived credibility - increased inter-personal trust - improved communication skills - reduced hyperactivity - emotional symptoms - conduct problems - peer problems Hyperactivity levels decreased significantly in adolescent control group. CBI had more positive effect on adolescent girls than boys. No significant gains observed among adolescent boys age 12–16 years	Strong	
**Psychosocial Structured Activities (PSSA) intervention**[49] Uganda *Ager et al., 2011*	Displaced children aged 7 – 12 years in primary schools in Uganda	PSSA intervention, school-based multi-phased approach designed to enhance resilience, coping skills, self esteem and future planning through structured activities- play therapy, art, drama 15 × 60 min sessions delivered over course of five weeks. Implemented by trained school teachers	PSSA intervention builds upon work of CBI intervention implemented in Palestine [50]. Intervention implemented previously in US and Indonesia PSSA encourages parental involvement through periodic meetings.	Quasi experimental N = 403 primary school students (mean age 10.23 years) from 12 schools (8 intervention) in Uganda 12 month follow up	Significant improvement in participants’ wellbeing, as measured by parents and children (but not teacher). Evidence from parent and teacher report of girls making greater progress than boys Evidence of older children making greater progress than younger children.	Moderate	
**Teaching Recovery Techniques (TRT) intervention for war affected children**[50] Gaza, Palestine *Quota et al., 2012*	War affected children age 10–13 years in Palestine	TRT intervention aims at creating safety and feelings of mastery, and incorporates trauma-related psychoeducation, CBT methods, coping skills training 16 sessions implemented over 4 weeks after school (two weekly 2 hour sessions) Programme implemented by psychologists	Evidence based intervention [51-53] Programme implemented by psychologists as an extra curricular activity on school premises. Families involved through homework activities	RCT N = 722 children age 10–13 years from four schools assigned to intervention and control group Six months follow up Control received normal school-provided support.	Intervention significantly reduced proportion of clinically significant Post-Traumatic Stress syndrome at post-intervention. No programme effect for girls.	Strong	
					Girls significantly benefited from intervention (in symptoms and proportion of clinically significant PTSS) if they showed low peritraumatic dissociation.		
**Classroom-based group intervention for children exposed to war**[54] Lebanon *Karam et al., 2008*	War affected children age 6–18 years in Lebanon	Intervention consisted of cognitive behavioural strategies and stress inoculation training 12 × 90 min sessions implemented over 12 consecutive school days	Intervention delivered by teachers Teachers received one day training and supervised every 2–3 sessions Study used only diagnostic assessment measures	Quasi experimental N = 209 students (mean age 11.7 years) from six schools Matched control group did not receive structured activities	No significant effect of the intervention of rates of major depressive disorder, separation anxiety disorder and post-traumatic stress disorder. Rates of disorders peaked one month post-war and decreased over one year. Post-war major depressive disorder, separation anxiety disorder and post-traumatic stress disorder were associated with pre-war SAD and PTSD scores, family violence parameters, financial problems and witnessing war events.	Strong	
**Writing for Recover (WfR) intervention**[55] Gaza *Lange-Nielsen et al., 2012*	Adolescents age 12–17 from refugee camp in Gaza	Manual based short term writing intervention involves adolescents undertaking unstructured and structured writing detailing their traumatic memories and insights from what they have experienced Six short writing sessions over 3 consecutive days (2x15 min session each day).	Programme implemented by teachers who have received 1 day training 88.4% of participants reported participation as a positive experience at T3 and 94.3% at T4. Lack of adherence to manual reported	RCT N = 139 adolescents age 12–17 years from six schools Waitlist control Four-five month follow up	Significant decline in PTSD symptoms in both intervention and control group. Significant increase in intervention groups’ depression symptoms from T1-T2. Significant decline in depression symptoms from T3-T4. No significant change in intervention groups’ anxiety scores from T1 – T2 or T3 to T4.	Strong	
**Child-focused intervention for children living in conflict areas**[56] Palestine (Gaze and West Bank) *Loughry et al., 2006*	Children and adolescents (age 6–17 years) and their parents living in areas of conflict	Interventions aims to provide structured activities that support the resilience of children in conflict Intervention implemented over one year and focused on participation in recreational, cultural and other non-formal activities. Included parental involvement	Children’s activities included after-school recreation activities in community setting (e.g. summer camps) Activities for children’s parents included information classes and opportunities to join with children in structured activities	Quasi experimental N = 400 children and adolescents Control group did not receive structured activities	Significant reduction in intervention groups’ - total problem scores - externalizing problem scorers - internalizing problem scores Intervention had some effect in improving parental support in West Bank children only.	Strong	
**Community-Based Interventions**							
**Study Name, Country, Study author**	**Target Group**	**Type of Intervention & Duration**	**Implementation Issues**	**Study design**	Outcomes*	**Effect Sizes**	**Quality Assessment**
**Population based intervention to promote youth health**[57] Goa, India *Balaji et al., (2011)*	Youth age 16-24	Community based intervention designed to promote youth health Intervention implemented over 12 months and consists of 3 main components: (i) Peer Education (ii) Teacher Training (iii) Health information Intervention implemented by intervention team which consisted of social worker, two psychologist and three peer educators	Community actively involved in programme planning Difficulties noted in the integration of peer education within existing school structures Community peer education was feasible and acceptable in rural community but not the urban community	Exploratory controlled evaluation N = 1803 students from two urban and rural communities Control communities were wait listed. 18 months follow up	Significant: - decrease in probable depression score (rural & urban) - greater knowledge and attitudes about emotional health (rural) - lower levels of suicidal behaviour (urban) Peer leaders reported increase in skills: - self-confidence - leadership ability - stress managements - conflict resolution - anger management and Improved student-teacher relationship post-intervention Significant: - increase in attitudes about reproductive and sexual health (rural & urban) - decrease in perpetration of physical violence (rural & urban) and substance abuse (urban) - Rural sample reported significant: - fewer menstrual complaints - higher levels of help- seeking for reproductive and sexual health problems by women Urban sample reported significant lower levels of - sexual abuse - RSH complaints - menstrual complaints	Not reported	Strong
**Familias Fuertas**[58] Honduras *Vasquez et al., 2010*	Parents and their 10–14 year old adolescents	Evidence based family skills building training programme. Focused on promoting consistent discipline, parental monitoring and positive communication patterns 7 activity based sessions Local nurses trained as FF facilitators	Programme is based on the evidence based “Strengthening Families” programme in US	Quasi-experimental design N = 41 parent-adolescent pairs Control received informational brochures 12 months follow-up	Significant improvement in intervention groups’: - positive parenting behaviours - positive perceptions among parents about their family relationships - parental self esteem Non significant reduction in adolescent or family member drug alcohol or tobacco use.	Not reported	Moderate
**Ishraq Programme**[59] Egypt *Brady et al., 2007*	Out of school adolescent girls age 13-15	Multidimensional community based programme aimed at improving girls’ life skills, functional literacy, recreational opportunities, health knowledge and attitudes and mobility and civic participation. Girls meet four times a week for 30 months in in groups of around 25 girls Programme implemented by ‘Promoters’ – young local women (age 17–25) trained in their role.	Important part of the programme was work carried out with brothers and other male relatives in helping them to think and act in a more gender equitable manner	Quasi-experimental N = 587 adolescent girls from four villages in Upper Egypt	Significant improvement in social participation. Girls in the programme significantly more likely - to know about key health and rights issues - to score higher on gender role attitude index - to make and keep friends Full term participants showed greatest increase in academic skills. Strong association between desire to delay marriage and participation in Ishraq.	Not reported	Moderate
**Stepping Stones**[60] South Africa *Jewkes et al., 2008*	Men and women age 16-23	HIV prevention programme aims to improve sexual and emotional health by developing strong, more equal relationships Programme delivered to single sex groups. Programme lasts 50 hours over 6–8 weeks	Workshops cover relationship skills, including assertiveness training as well as information of STIs and condoms. Facilitators were the same sex as the participants. Programme generally run on school premises after school hours	Cluster RCT N = 2776 men and women age 15–26 years Two year follow-up	Reduced (but not significant) levels of depression reported in men at 24 month follow up. No significant change in women’s depression levels in intervention group. Significant reduction in male: - physical and sexual partner violence (two year follow up) - problem drinking (one year follow up) - number of HSV-2 infections over 2 years No evidence of desired behaviour change in women. No evidence of lowered incidence of HIV.	Not reported	Strong
**The Collaborative HIV Adolescent Mental Health Programme South Africa (CHAMPSA)**[61] South Africa *Bell et al., 2008*	Adolescents (4^th^ and 5^th^ grade) and their families	HIV prevention programme aims to strengthen family relationships as well as target peer influences 10 (90 minute) sessions delivered by community caregivers over 10 weekends to families	CHAMPSA is an adaptation version of the evidence based CHAMP Family programme [62] Community members involved in programme design, delivery and research Families paid $8 for each session attended	RCT N = 478 families rearing 579 children Control received existing school based HIV prevention curriculum	Significant increase in caregivers’: - communication skills - monitoring of children - social primary networks	Caregiver data: HIV transmission knowledge ES = 0.631 Less stigma toward HIV infected people ES = 0.403 Caregiver monitoring 3 family rules ES = 0.307 Caregiver communication comfort ES 0.407 Caregiver communication frequency ES = 0.197 Social networks – primary ES = 0.265 Child data: AIDS transmission knowledge ES = 0.496 Less stigma towards HIV infected people ES = 0.698	Strong
**South Africa’s Intervention with Microfinance for AIDS and Gender Equity: IMAGE study**[63] South Africa *Kim et al., 2009*	Women age 18 years and over	Community based combined gender and HIV training programme and microfinance initiative aims to address gender roles, poverty self-esteem, communication, domestic violence and HIV Delivered over 12–15 months. Phase 1 (6 months) consisted of 10 training sessions. Phase 2 encouraged wider community mobilization to engage youth and young men in the intervention	Microfinance only intervention provided women with small loans to women IMAGE ‘Sisters for Life’ gender and HIV training programme integrated gender and HIV training programme into fortnightly microfinance meetings The addition of a training component to group-based microfinance programmes may be critical for achieving broader health benefits	Cluster randomized trial Three randomly selected matched clusters (i) four villages with 2 year exposure to IMAGE combined with microfinance (ii) four villages with 2 year exposure to microfinance and (iii) control villages not targeted by any intervention. N = 860 female loan recipients enrolled	Significant improvements in empowerment among women in combined IMAGE microfinance group Micro finance only group showed no improvements in empowerment. Significant improvements in: - intimate partner violence (IPV) and HIV risk behaviour (women in combined IMAGE - microfinance group) - economic wellbeing (women in microfinance only and combined group) Micro finance only group showed no improvements in IPV and HIV risk behaviour	Not reported	Moderate
**IMAGE and microfinance study**[64] South Africa *Pronjk et al., 2006*	Women in rural areas in South Africa (age 14–35 years)	Community based combined gender and HIV training programme and microfinance initiative aims to address gender roles, poverty self-esteem, communication, domestic violence and HIV (same as above)	Programme consists of poverty-focused microfinance initiative and a 12–15 month participatory ‘Sisters for Life’ gender and HIV training programme	RCT N = 3339 women from 8 villages Two year follow up	Programme participants reported: - 55% fewer acts of violence by their intimate partners in previous 12 months - fewer experiences of controlling behaviour by their partners - increased economic wellbeing among intervention group Significant higher levels of social participation	Not reported	Moderate
**SUUBI - economic empowerment intervention**[65-68] Uganda *Ssewamala et al., (2009a, 2012, 2010, 2009b)*	AIDS-orphaned children in final year of primary school	Economic intervention that involves creating asset-building opportunities and promotion of life options by providing (i) 1–2 hour workshops focused on asset building and future planning (ii) monthly mentorship programme for adolescents with peer mentors on life options (iii) Child Development Account dedicated to paying for secondary schooling, vocational training and/or family small business	Girls were likely to have higher self-esteem than boys Homeownership was significantly associated with positive changes in children’s self-esteem Children in treatment group saves, on average an equivalent of USD$6.33 a month or UDS$76 a year	RCT N = 267 children from Grade 7 in 15 primary schools Control group received usual care for orphaned children Ten month follow up	Significant - increase in self-rated self esteem at 10 months post-intervention - decrease in depression - increase in academic performance educational - aspirations and attitudes towards sexual risk taking behaviour - reduction in sexual risk taking intentions - increase in self rated physical health functioning	Not reported	Strong

* Significance levels p ≤ 0.05.

## Results

**Figure 1:**
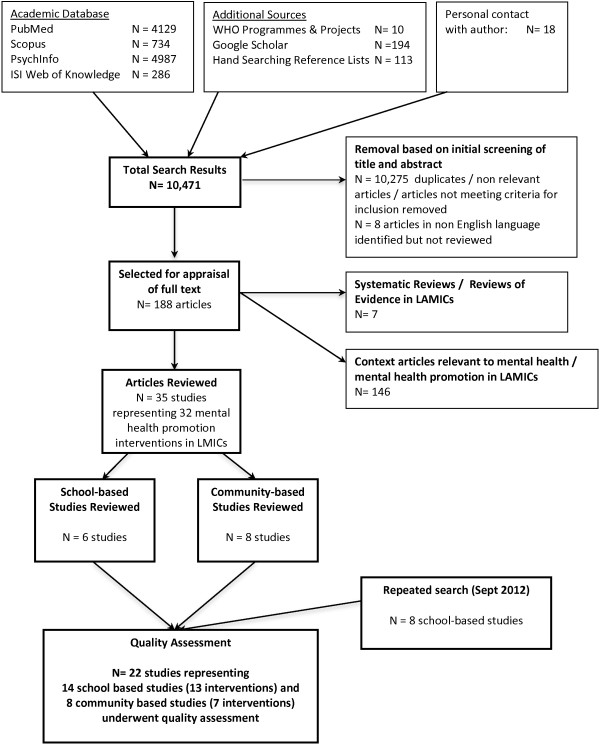
Search results from original search of mental health promotion interventions in LMICs.

The results of the search and study selection are shown in Figure 1. The original search process carried out in 2011 produced 10,471 articles, 188 articles of which were selected for full review and exported to Endnote. Of these, 146 were either contextual articles related to mental health promotion in LMICs or studies that did not meet one of our inclusion criteria. Seven articles were systematic/summary reviews of the evidence base in LMICs, five of which were reviews of interventions for young people. A total of 35 primary studies were selected for review. Of these, 14 studies evaluated school or community-based interventions for young people in LMICs. During the repeated search performed in September 2012, a further eight studies evaluating school-based interventions were identified. The combined searches resulted in a total of 22 studies (14 school and eight community-based studies) undergoing quality assessment. No studies in non-English language specific to school and community based-interventions were identified in the review process.

The five systematic review articles from LMICs that were identified examined the effectiveness of HIV related lifeskills interventions [69,70] and psychosocial interventions for children and adolescents affected by armed conflict in LMICs [71-73]. All relevant interventions across the reviews were identified and cross-referenced with the primary articles retrieved through the electronic search. Given the specific focus of this systematic review on mental health promotion and primary prevention, several studies from these systematic reviews did not meet the inclusion criteria for this review.

Regarding the number and percentage of evaluation studies carried out across LMICs, 18.2% (N = 4) of the interventions were carried out in low income countries, 36.4% (N = 8) were carried out in lower middle income countries and 45.4% (N = 10) were carried out in upper middle income countries. Just under one third of the interventions (N = 7) were carried out in South Africa alone.

### School-based programmes

Fourteen studies describing thirteen interventions implemented in school settings in eight LMIC countries were identified. Four studies were carried out in Gaza/Palestine [48,50,55,56], three were carried out in South Africa [37,38,41,44], two in Uganda [45,49] and one intervention was carried out in India [35], Chile [36], Mauritius [42], Nepal [47], and the Lebanon [54]. The majority of studies (>60%) were published between 2010–2012. The quality of evidence from the majority of studies was strong. A total of eight studies received a strong quality rating [42,45,47,48,50,54-56], five studies received a moderate quality assessment rating as a result of selection bias [36,44] and not reporting the percentage of withdrawals/dropouts [35,38,49]. One study received a weak quality assessment rating due to selection bias, not reporting confounders and not reporting level of withdrawals [41].

The programmes were mental health promotion and universal prevention interventions designed for all children and adolescents of school going age. Interventions varied slightly in their focus from the development of social, emotional, problem solving and coping skills [35,41] to a combined mental health promotion with physical fitness programme [36], combined mental health promotion and sexuality education [37,38] and a universal depression prevention intervention [42]. Two interventions were designed specifically to support AIDS orphaned children, one was an art intervention [44], another was a peer support intervention led by teachers [45]. Seven interventions (eight studies) were school-based psychosocial interventions implemented in countries affected by armed conflict [47-50,54-56]. These interventions were designed to reduce distress, enhance resilience and coping skills. Four of these interventions incorporated cognitive behavioural techniques (CBT) and trauma related psychoeducation modules [47-50,54]. One intervention consisted of short writing sessions [55], another provided structured recreational activities [56].

Seven of the school-based interventions were designed for post-primary school students (>12 years of age). Four interventions were implemented with a broad age range from 6 – 18 years [44,48,54,56]. Three interventions were implemented with children in the senior end of primary school (>10 years of age) [45,49,50]. Eight interventions were implemented by the class teacher [35-38,42,44,49,54,55], with the remaining interventions implemented by mental health professionals [41,48,50], locally trained paraprofessionals [47] and local youth workers [44,56]. The majority of session ranged in length from 11 – 16 sessions implemented weekly. One intervention provided six booster sessions at 12 months following completion of the programme [37,38]. Eight school interventions were developed in the implementing country. Five interventions were adapted versions of evidence-based interventions from high income countries [37,38,42,47-49].

Regarding intervention outcomes, in terms of the seven universal programmes implemented with children affected by armed conflict, the findings are generally positive but with some studies reporting mixed effects. Loughry et al. [56] reported that the after-school recreational activities implemented over one year had a significant positive impact on children and adolescents’ externalising and internalising problem scores and also improved parental support as a result of parental involvement in the structured activities. Khamis et al. [48] reported that the Classroom-Based Intervention (CBI) had a significant positive effect on children (age 6–11) and adolescents (age 13–16) in terms of improved social and emotional wellbeing, communication skills and reduced conduct and peer problems and hyperactivity levels. Ager et al. [49] reported similar findings for the school-based Psychosocial Structures Activities intervention (PSSA) which is based on principles of the CBI, with the intervention having a significant positive effect on primary school children’s (mean age 10 years) wellbeing. Interestingly, the CBI study carried out in Nepal reported specific gender effects, with significant reductions in psychological difficulties and aggression among males only and improved prosocial behaviour among females only [47]. Two studies reported less positive findings. Karam et al. [54] found that the cognitive behavioural therapy intervention (CBT) implemented over 12 consecutive days had no significant effect on participant rates of depression, separation anxiety and post-traumatic stress disorder (PTSD). Lange-Nielsen et al. [55] reported that the three day short-term writing intervention had no effect on participants’ PTSD symptoms and anxiety scores. This study also reported that the writing intervention lead initially to significantly increased depression symptoms for participants between pre and post-intervention but that symptoms significantly declined at five months follow up. Contrasting findings in terms of gender effects were reported across three studies; two studies reported that the interventions have a more positive effect on girls [48,49] while another intervention reported no programme effect for girls with PTSD scores improving only in male participants [50].

Regarding the universal lifeskills and resilience school-based interventions, all six studies reported significant positive effects on students’ mental health and wellbeing in terms of improved self-esteem [35,36,42], motivation [38] and self-efficacy [44]. The peer-group support intervention implemented with AIDS orphan children resulted in significant improvements in participants’ depression, anger and anxiety scores but not for self-concept [45]. The combined fitness lifeskills education intervention reported improvements in anxiety symptoms, however, there was no change in participants’ depression scores [36]. The depression prevention intervention on the other hand reported a significant reduction in depressive symptoms (medium effect size) and hopelessness (medium effect size) and a significant increase in coping skills (medium effect size) amongst participants in the intervention group [42]. Long-term findings from this depression prevention intervention included improved self-esteem and coping skills (medium effect size) at six months follow up. In addition, the resilience intervention in South Africa also reported long-term findings with improved self-appraisal scores maintained at three months follow up [41]. Additional outcomes from these studies include improved behaviour [35], school adjustment [35], fitness [36], attitudes about reproductive and sexual health [37] and a reduction in the level of substance misuse [37]. The art intervention for AIDS orphan children reported the least positive findings with a significant improvement reported in the intervention groups’ self-efficacy score but no change in participants’ depression, self-esteem and emotional and behavioural scores [44]. While the results from the resiliency intervention indicated significant improvements in participants’ emotional reactivity, self appraisal and interpersonal strength, the weak quality of this study must be considered when interpreting these findings.

### Community-based interventions

This review identified eight studies evaluating seven out-of-school community interventions for adolescents in five countries. Four studies were carried out in South Africa [60,61,63,64], one study was carried out in India [57], Honduras [58], Egypt [59] and Uganda [65-68]. All eight studies were published between 2006 and 2010. The quality of evidence from these studies was moderate to strong. Four studies received a strong quality assessment rating [57,60,61,65] and four studies received a moderate quality assessment rating due to small sample size [58] and failure to report validity and reliability of measures used in three studies [59,63,64].

Interventions included a multi-component school and community-based intervention for youth aged 16–24 years [57]; a family-based strengthening programme (*Familias Fuertas*) for parents and their adolescent children [58]; a multidimensional programme (*Ishraq*) aimed at improving the life skills, literacy, recreational activities and health knowledge of 13–15 year old girls in Egypt [59] and combined HIV prevention and lifeskills interventions (*Stepping Stones* and *CHAMPSA*) for adolescents in South Africa [60,61]. Two studies evaluated the Intervention with Microfinance for AIDS and Gender Equity (*IMAGE*), a poverty-focused microfinance initiative for women that is combined with a 12–15 month gender and HIV education curriculum [63,64]. One study examined the effects of small individual loans and mentorship on health and mental health functioning of primary school children [65-68]. Five of the seven interventions were designed for young people aged 13+. The Familias Fuertas intervention was designed for children age 10–14 and one of the evaluations of the IMAGE microfinance intervention was implemented with females aged 18 and over. Two interventions provided parent training [58,61] and two interventions were designed specifically for females [59,63,64]. Five of the interventions were implemented by local trained community caregivers [59-61,63-68]. The Familias Fuertas intervention was implemented by a local nurse [58] and the multi-component school and community intervention in India was implemented by a team of social workers, psychologists and peer educators [57]. Five of the interventions were developed in the implementing country. Two interventions were adapted versions of evidence-based interventions that were developed in the United States [58,61].

Collectively, the results from these studies indicate the significant positive effect of community-based mental health promotion interventions on young people’s mental health and social wellbeing. Five interventions provided strong evidence of their positive impact on mental health. Balaji et al. [57] reported that the community-based youth health intervention in India resulted in significant improvements in participants’ depression scores, reported levels of suicidal behaviour, and knowledge and attitudes about mental health. South Africa’s IMAGE intervention resulted in significant improvements in empowerment, social participation and levels of openness among women in the combined IMAGE-microfinance intervention, with no change evident the microfinance only intervention [64]. In addition, Pronijk et al. [64] reported that participants in the IMAGE intervention were significantly more likely to participate in training, and had greater participation in social and community groups. Ssewamala et al. [65,66] reported that the SUUBI economic empowerment intervention for AIDS orphaned children had a significant positive impact on participants’ self-esteem and levels of depression. Results from the parent-youth interventions indicate the significant effect of the programmes on positive parenting communication and behaviours, parental self-esteem and family relations [58,61].

Other reported outcomes also included significantly improved: peer relations [59]; academic performance [59,68]; student-teacher relations [57]; communication [64]; improved gender roles [64] and significantly reduced sexual risk behaviour [60,63] one year follow up; physical and sexual partner violence [57,60,63,64]; and substance abuse [57,60]. Bell et al., [61] reported medium effect sizes for improved caregiver communication comfort. Long-term findings from the *Stepping Stones* intervention include reduced physical and sexual partner violence at two years follow- up, and reduced substance abuse at one year follow up [60].

## Discussion

This review sought to determine the effectiveness of mental health promotion interventions designed for young people (aged 6–18 years) in LMICs. A total of 22 studies evaluating 20 interventions were identified. The majority of interventions were implemented in upper and lower middle income countries, thus highlighting the paucity of evidence from low income countries. Four interventions were carried out in low income countries, three of which were conducted in Uganda. It is encouraging to note, however, the significant increase in publications from LMICs in the last four years, with the majority of interventions identified in this review published since 2008.

With regard to the school-based interventions, the quality of evidence from the 14 studies is moderate to strong. Findings from these studies indicate that there is reasonably robust evidence that school-based programmes implemented across diverse LMICs can have significant positive effects on students’ emotional and behavioural wellbeing, including reduced depression and anxiety and improved coping skills. Promising interventions include the Resourceful Adolescent Program (RAP-A), which was implemented by teachers in Mauritius [42]. This study is an example of an evidence-based intervention adapted from a HIC for implementation in a LMIC and points to the potential of such interventions when adapted to meet the cultural needs of young people in LMICs. Another promising intervention is the teacher led peer-group support intervention for AIDs orphaned children which was implemented in a low-income country [45]. The findings from this study suggest the potential of peer support mental health promotion interventions in optimizing adjustment and decreasing the psychological distress associated with AIDS orphanhood in the adolescent age group. Such interventions may have great potential in addressing the increased risk of depression, peer relationship problems, post-traumatic stress and conduct problems among AIDS orphans [74-76]. There is also some encouraging evidence that interventions which combine lifeskills with reproductive and sexual health education [37,38] and physical health and fitness [36] can have a significant positive effect on pupils’ risk-taking and prosocial behaviour. These findings are consistent with the substantive evidence from multiple reviews of school-based interventions in HICs which report the greater effectiveness of multi-component interventions (i.e. interventions that adopt a social competence approach and develop supportive environments), when compared with interventions that focus on specific problem behaviours [8,28,77-79]. The integration of multicomponent programmes within a whole school approach [13] based on generic social and emotional skills training addressing comon risk and protective factors, delivered within a supportive school environment in partnership with parents and the local community, has the potential to reach larger population groups with fewer resources.

The evidence for universal interventions implemented with young people affected by war attests to the important role of the school as an accessible setting for such interventions. Similar to previous reviews [72,73], the heterogeneity across the studies in terms of programme content, delivery, duration, and study sample makes it difficult to draw general conclusions about the effectiveness of these interventions as a whole. However, there is evidence that the more structured interventions of longer duration can have a significant positive effect on mental health and wellbeing. The results from the Classroom-Based Intervention (CBI) and the school-based Psychosocial Structured Activities intervention (PSSA), which is based on CBI principles, highlight the positive effect of these interventions on young people’s social, emotional and behavioural wellbeing. The differential effects according to gender reported across these interventions, however, calls for further investigation into possible gender specific components. The optimum age for programme implementation also needs further examination. There is evidence from Khamis et al. [48] that CBI did not yield the same significant positive changes with older males (12–16 years) as with the younger group (aged 6–11). This finding is in line with substantive evidence from HICs regarding the need to reach children when they are young in order to sustain their existing resilience and strengthen their coping capabilities [4,12,80-82].

Non-significant findings were also found for a writing intervention implemented with young people aged 12–17 [55] and a CBT intervention implemented with children and adolescents aged 5–16 [54]. It is important to note the initial negative impact of the writing intervention on participants’ depression symptoms, which then subsequently declined at follow-up. Common characteristics of these interventions were their short duration and the broad age range of the intervention participants. This is in contrast to the year long after-school intervention implemented with children and adolescents and their parents living in Gaza and the West Bank, which resulted in significant improvement in participants’ social and emotional wellbeing and parenting behaviours [56]. The results from these studies underscore the importance of understanding optimum programme components in terms of content, duration, and target age range in order to ensure the development of effective school-based interventions in conflict areas. This is in line with recommendations from previous reviews of school-based interventions implemented in war exposed countries [71,72] including those from secondary prevention interventions, not covered in this review, which also point to the need for more rigorous research on the differential intervention effects related to age, gender and war-related experiences [73,83]. The studies in this review support previous findings concerning the role of universal school programmes for children living in conflict areas as an effective, accessible and efficient means of enhancing and protecting good mental health alongside more targeted approaches for students at higher risk [84]. The exploration of a whole school approach to interventions in this area carries potential for reaching the wider community through the school setting.

The majority of the school-based interventions included in this review were implemented with young people age 12–16 years. In view of the paucity of evidence of mental health promotion interventions for young children in primary schools in LMICs (age 5–10 years), there is an urgent need for high quality studies with longitudinal designs to assess the impact of school-based intervention for younger primary school children in order to strengthen the evidence base in this area. Schools are arguably one of the most important settings for reaching out to young children and their families and early intervention is recognised as one of the key principles of effective mental health promotion and prevention interventions [4,8,12,80]. In addition, eight of the interventions were implemented by trained class teachers, with the remaining interventions implemented by psychologists, paraprofessionals and youth workers. As Srikala & Kumar [35] argue, any programme incorporated into the education system in LMICs has to be feasible and cost-effective. The findings from this review suggest that trained teachers can effectively deliver mental health promotion interventions. Similar to findings from HICs, several of the studies reviewed highlighted the importance of teacher training and the provision of ongoing support during programme implementation. Harnessing the skills of teachers and providing support in the school setting offers a sustainable and low cost method of improving children’s emotional and behavioural wellbeing, developing positive coping strategies and promoting school performance. As the Millennium Development Goals have set out as a target that all boys and girls will be able to complete a full course of primary schools by 2015, the integration of social and emotional learning and lifeskills development in the primary school curriculum and the development of a whole school approach to health promotion is an important component of this development agenda.

In terms of the evidence for community-based interventions in LMICs, there are a limited number of very promising youth interventions addressing sexual and emotional health, HIV prevention, substance misuse, violence prevention, functional literacy, economic empowerment and social participation among excluded groups. The results from these multicomponent interventions are impressive given the improvements that were shown across a broad range of adolescent health outcomes. Although limited in number, the three microfinance interventions for young adults and primary school children included in this review, provide encouraging evidence that combined microfinance and training interventions promoting essential lifeskills, asset building and reourcefulness, can result in significant mental health and wellbeing benefits. Further evaluations of such multicomponent community-based interventions are needed to determine the long-term impact on more specific mental health outcomes.

### Study limitations

This systematic review has a number of important limitations, which impact on its validity. Firstly, there are limitations relating to the scope of the systematic search, which impact on the validity of the findings. Due to the timescale and resources available, a systematic search for studies published in the grey literature was not included, and neither was effort made to find well-designed studies that had not been reported at all due to non-significant findings. Furthermore, a search in languages other than English was not undertaken and, therefore, key studies in the other former colonial languages of French, Spanish, Portuguese and Dutch were not included.

Secondly, there are limitations relating to the selection criteria, which also impact on the validity of the findings. Studies not employing traditional experimental or quasi-experimental designs were excluded from the search and therefore, qualitative and other such study designs were discarded in the search process. Of the studies that were included, justification of sample size and validation of the outcome measures employed were not reported in a small number of the papers. It could be argued that such studies should also have been excluded from the review, but in our methodology they were included but received lower quality assessment ratings due to the absence of information on these issues. Finally, as a narrative synthesis the review is not designed to generate summary statistics derived from meta-analyses. Despite these limitations, the studies included in this review clearly demonstrate that high quality and effective mental health promotion interventions, and their evaluation through well-designed research studies, are feasible in LMIC settings.

## Conclusions

The review findings indicate that mental health promotion interventions for young people can be implemented effectively in LMIC settings. There is good quality evidence regarding the impact of school-based programmes and promising evidence from multicomponent community-based studies that such interventions offer a viable means of promoting the mental health and wellbeing of young people. Notably, the studies reviewed demonstrate the feasibility and effectiveness of integrating mental health promotion interventions into education and community programmes such as community empowerment, poverty reduction, HIV/AIDS prevention, reproductive and sexual health. While the mental health promotion interventions identified in this review have achieved success across a diverse range of countries, relatively few have been systematically scaled up to serve the needs of young people at a regional or national level. Thus, evidence for their sustainability and effectiveness when scaled up through the educational system and community settings in LMICs needs to be strengthened, especially in low-income countries. In addition, the short-term follow-up periods of many of the studies point to the need for future research to evaluate long-term outcomes. Research is also needed to strengthen the evidence-base on the interrelationship between mental health and other health, educational and social wellbeing outcomes. Such research would strengthen the case for mainstreaming the integration of mental health into key health, education and development priorities for young people in LMICs.

The studies reviewed demonstrate the feasibility and potential sustainability of implementing mental health promotion interventions in LMICs through employing existing infrastructures and resources, working with local teachers, community workers, young people and their families. Further research is needed on the contextual factors influencing the adoption and adaptation in LMICs of well-validated interventions that have been developed in low resource settings in HIC countries. In particular, implementation research is needed to ensure the successful adaptation and transfer of school-based interventions for younger primary school children across educational, cultural and socio-economic settings. The development of culturally valid measures of mental health, that will support the evaluation of culturally appropriate interventions in LMICs, is also identified as an area for methodological development. Existing standardized mental health measures from HICs need to be locally validated and the development of culturally sensitive indicators of positive mental health and wellbeing will be particularly important in determining the benefits of mental health promotion interventions delivered in diverse cultural contexts. Developing capacity in LMICs for the implementation and evaluation of mental health promotion policies and practices is fundamental to promoting and sustaining action for positive youth mental health development.

## Competing interests

The authors declare that they have no competing interests.

## Authors’ contributions

MB designed the study and AC performed the data search; MB and AC reviewed the studies and carried out the quality assessment ratings; RJ and VP contributed to the interpretation of the data and the drafting of the manuscript. All authors read and approved the final manuscript.

## Pre-publication history

The pre-publication history for this paper can be accessed here:

http://www.biomedcentral.com/1471-2458/13/835/prepub
